# Splicing alterations in pancreatic ductal adenocarcinoma: a new molecular landscape with translational potential

**DOI:** 10.1186/s13046-023-02858-z

**Published:** 2023-10-26

**Authors:** Emilia Alors-Pérez, Sergio Pedraza-Arevalo, Ricardo Blázquez-Encinas, María Trinidad Moreno-Montilla, Víctor García-Vioque, Inmaculada Berbel, Raúl M. Luque, Bruno Sainz, Alejandro Ibáñez-Costa, Justo P. Castaño

**Affiliations:** 1 Maimonides Biomedical Research Institute of Córdoba (IMIBIC), Cordoba, Spain; 2 Department of Cell Biology, Physiology, and Immunology, University of Córdoba, Cordoba, Spain; 3 Reina Sofía University Hospital (HURS), Cordoba, Spain; 4 Centro de Investigación Biomédica en Red de Fisiopatología de la Obesidad y Nutrición, (CIBERObn), Córdoba, Spain; 5 Department of Cancer Biology, Instituto de Investigaciones Biomédicas Alberto Sols CSIC-UAM, Madrid, Spain; 6 Cancer Stem Cells and Fibroinflammatory Microenvironment Group, Instituto Ramón y Cajal de Investigación Sanitaria (IRYCIS), Area 3, Cancer, Madrid, Spain; 7 Gastrointestinal Tumours Research Programme, Biomedical Research Network in Cancer (CIBERONC), Madrid, Spain

**Keywords:** Pancreatic cancer, Splicing, Splicing machinery, Splicing isoforms, Splicing modulation

## Abstract

Pancreatic ductal adenocarcinoma (PDAC) remains one of the most lethal cancers worldwide, mainly due to its late diagnosis and lack of effective therapies, translating into a low 5-year 12% survival rate, despite extensive clinical efforts to improve outcomes. International cooperative studies have provided informative multiomic landscapes of PDAC, but translation of these discoveries into clinical advances are lagging. Likewise, early diagnosis biomarkers and new therapeutic tools are sorely needed to tackle this cancer. The study of poorly explored molecular processes, such as splicing, can provide new tools in this regard. Alternative splicing of pre-RNA allows the generation of multiple RNA variants from a single gene and thereby contributes to fundamental biological processes by finely tuning gene expression. However, alterations in alternative splicing are linked to many diseases, and particularly to cancer, where it can contribute to tumor initiation, progression, metastasis and drug resistance. Splicing defects are increasingly being associated with PDAC, including both mutations or dysregulation of components of the splicing machinery and associated factors, and altered expression of specific relevant gene variants. Such disruptions can be a key element enhancing pancreatic tumor progression or metastasis, while they can also provide suitable tools to identify potential candidate biomarkers and discover new actionable targets. In this review, we aimed to summarize the current information about dysregulation of splicing-related elements and aberrant splicing isoforms in PDAC, and to describe their relationship with the development, progression and/or aggressiveness of this dismal cancer, as well as their potential as therapeutic tools and targets.

## Introduction

### Pancreatic ductal adenocarcinoma

Pancreatic ductal adenocarcinoma (PDAC) is the most common and aggressive type of tumor in the pancreas (80%). PDAC mortality rate is one of the highest of all cancers worldwide, and it is even higher in Europe and North America. It accounts for 4.7% of all cancer-related deaths, almost matching the number of new cases. According to a study involving 28 European countries, it is projected to surpass breast cancer as the third leading cause of cancer death by 2025 [1]. Low survival rates are associated with late diagnosis, the presence of metastasis and the development of drug resistance. PDAC risk factors include age, genetics (in around 10% of cases), tobacco and alcohol use, pancreatitis, and obesity, among others, which generally increase inflammatory pancreatic damage [2].

PDAC develops from pre-invasive lesions. These include cystic lesions such as intraductal papillary mucinous neoplasm (IPMN), mucinous cystic neoplasm (MCN), and intraductal tubulopapillary neoplasm (ITPN). However, the most common lesion is pancreatic intraepithelial neoplasia (PanIN), which is non-cystic. Cystic lesions can be diagnosed using imaging methods, but PanINs cannot be detected early or through methods other than microscopic examination [3]. The progression of PanINs encompasses various grades, advancing towards significant dysplasia. This process is marked by the loss of cell polarity and an enlargement of the nucleus. The transformation of pancreatic tissue has been linked to various mechanisms, including genomic instability and mutations. Multiple studies have demonstrated the presence of some of the most prevalent mutations in PDAC within pre-invasive lesions such as PanINs [4]. For example, it has been shown that the frequency of *KRAS* mutation increases as the disease advances to a higher grade of dysplasia, but it even appears in the early stages of the lesion. *KRAS* is the most commonly mutated gene in PDAC (95% of patients) and the main driver of tumorigenesis, with G12D its most prevalent codon mutation [5]. Other frequent mutations have been characterized in PDAC, including mutations in genes encoding tumor suppressor proteins, like *CDKN2A*, *TP53* or *SMAD4*, or genes involved in essential cell processes, such as chromatin remodeling and DNA damage repair, likely contributing to increased genomic instability [6]. Another cause of DNA damage is telomere shortening, which seems to be an early event in pancreatic lesion. Nevertheless, although the genomic landscape of PDAC is well characterized and most frequent mutations have been proposed as therapeutic targets for PDAC on multiple occasions, current therapies in PDAC do not include these genetic alterations as targets [7, 8]. Along with the described genetic alterations, there are additional factors that reside at different hierarchical levels and contribute to PDAC malignancy. The histological/tissue features comprise a critical level in this tumor type because of the nature, volume, and cellular composition of the stromal compartment, increasing heterogeneity and hindering drug delivery. Likewise, the inflammatory component that accompanies pancreatic damage keeps developing throughout progression to adenocarcinoma. Besides cancer cells, PDAC contains several relevant cell types that comprise an intricate microenvironment, including different classes of fibroblasts, pancreatic stellate cells, cancer stem cells, macrophages, infiltrated lymphocytes, and vascular cells [9]. These cells have been shown to communicate with cancer cells, and their interaction is necessary for tumor progression, promoting tumor growth, angiogenesis, metastasis, and driving drug resistance [10].

Despite the remarkable advances achieved regarding the molecular makeup of PDAC, the number of patients who survive this pathology has only modestly improved in the last years, with a dismal 5year survival rate below 11–12%. Therapeutic approaches are limited, being surgery the only “curative” option, only effective in early diagnosed localized cases (15–20%). In the case of locoregional stage, neoadjuvant treatment may be used to make the tumor removable; while in the case of metastatic disease, surgery is not an option and only chemotherapy is offered. Moreover, chemotherapeutic treatments are limited, and although the latest combinations, including FOLFIRINOX (folinic acid, 5-fluorouracil, irinotecan, and oxaliplatin) or gemcitabine plus nab-paclitaxel, have extended progression-free survival, some treatment regimens are toxic and overall survival remains poor (< 12 months) [11]. For all these reasons, novel research avenues are being explored to develop alternative approaches, more effective treatments and early biomarkers [12]. In this context, the splicing process and its dysregulation has emerged as potential novel molecular tools to combat PDAC.

### The spliceosome and the splicing process

Splicing is a complex cellular mechanism by which the immature or precursor RNA is processed, removing the sequences that will not be part of the final RNA, or introns, and binding together the exons that form the mature RNA [13, 14]. However, most of the genes (> 95%) do not undergo this simple cut and paste process, also known as constitutive splicing, but rather they undergo an intricately regulated process, called alternative splicing [13, 15, 16]. This phenomenon allows the generation of different combinations of final sequences through the inclusion and exclusion of concrete groups of exons, which results in a variety of mature RNA transcripts from the same precursor, termed splicing variants or isoforms, that may carry out different or even opposite functions [17]. This is an essential cellular process that ensures an appropriate regulation of gene expression as it enables an increase in the variety of genes and thereby enhances the versatility of the genome [16]. For all these reasons, the accurate regulation of the splicing process is crucial for the correct development and homeostasis of the cell and the organism [18]. The process of splicing and its delicate regulation is carried out by the spliceosome, a ribonucleoproteic complex that recognizes specific RNA sequences to precisely localize the introns and cut them, and subsequently bind the adjacent exons [19]. In mammals, there are two different spliceosomes that act separately: the major spliceosome that processes more than 99% of the introns, and the minor spliceosome that acts over a small and specific set of introns [20]. Accordingly, introns are classified as U2-type (or -dependent, GT-AT) and U12-type (or -dependent, AT-AC), depending on the spliceosome that processes them or the flanking sequences [21]. Both spliceosomes consist of a main core of small nuclear RNAs (snRNAs), known as RNU1, RNU2, RNU4, RNU5 and RNU6 for the major spliceosome; and RNU11, RNU12, RNU4ATAC and RNU6ATAC (RNU5 is present in both), for the minor. These snRNAs are joined to proteins forming small nuclear ribonucleoproteins (snRNP; U1-U6) [19, 20]. In addition, the spliceosomes closely interact with the splicing factors, a diverse set of more than 300 molecules that complete the splicing machinery, helping the snRNPs to select and process the precise sequences, and taking part dynamically in every step of the process, participating in both general tasks as well as very specific events [22, 23].

The splicing process has been classically investigated in simple research models easier to study than human-based systems, like yeast, but the key steps are very well conserved in mammals. Summarizing the explanation by Matera and Wang in 2014 [24] and other studies [21, 25], U1 and U2 recognize and bind to 5’ and 3’ splice sites of the pre-mRNA, respectively. Next, U2 recognizes sequences in the so-called branch point and interacts with U1, forming the pre-spliceosome. In this step, the intron takes the form of a loop, which is called lariat. Then, the preassembled U4-U5-U6 complex is recruited, and several conformational changes take place to form a catalytically active complex, resulting in the U2/U6 structure that catalyzes the splicing reaction. In this step, U1 and U4 are released from the complex. At this point, the first catalytic step is carried out, cutting the binding between the first exon and intron-exon lariat intermediate. Finally, after some further conformational changes, the second catalytic step leads to the separation of intron and second exon and the binding of both exons, leaving the post-spliceosomal complex with the intron lariat free. Finally, U2, U5 and U6 are released. All the described steps are firmly regulated by several spliceosome proteins, which ensure that the cuts and bindings are correct, making possible the sequence recognition and putting together and separating the other components.

**Fig. 1:**
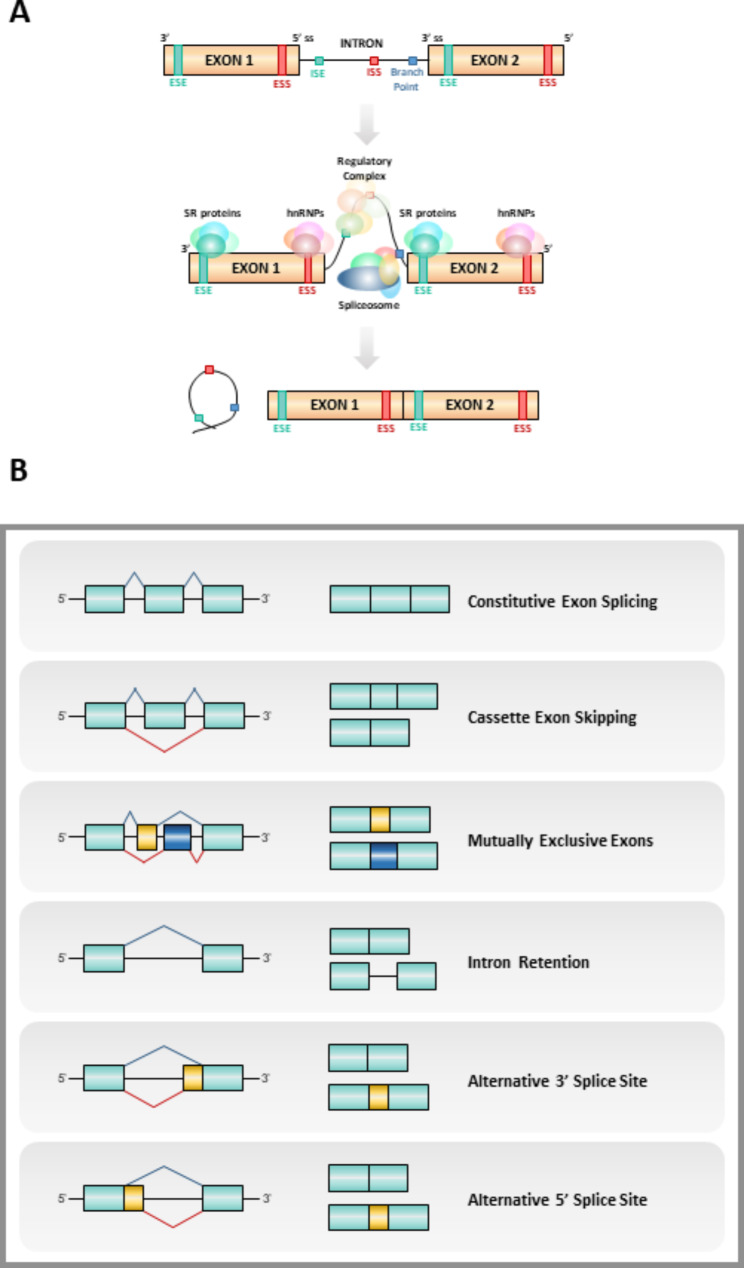
Splicing process. (**A**) Summary of the process of splicing, showing how the spliceosome machinery binds RNA in different regulatory sequences (exonic/intronic splicing enhancer [ESE/ISE] and exonic/intronic splicing silencer [ESS/ISS]), cuts the intron out and pastes the flanking exons together. (**B**) Different possibilities of alternative splicing events

Typically, the introns of mammals are long, and present several decoy splice sites that must not be spliced [15]. As mentioned earlier, alternative splicing is based on the inclusion/exclusion of selected sequences; therefore, a precise regulation is needed to correctly splice each sequence. To this end, cis-regulatory elements are distributed through the RNA, known as splicing regulatory elements, and, depending on their function and location, are classified in exonic/intronic enhancers/silencers (ESE, ISE, ESS and ISS, respectively) [15, 16, 26]. Those sequences recruit trans-regulatory elements, the splicing factors, which will suppress or activate steps of the splicing process (Fig. 1A). However, these events are completely dependent on the context, since the same factor may be a splicing enhancer and a splicing silencer if it binds to an enhancer or silencer element [16, 21, 26].

Furthermore, there are additional possibilities for splicing regulation. For instance, the structure of the precursor RNA may alter the accessibility to regulatory domains or even the spliceosome complexes [15]. In addition, the activity of the splicing machinery is finely regulated through modulation of its components, by gene expression regulation via transcription factors, miRNAs, epigenetics, etc [27–29], or posttranslational modifications, such as phosphorylation or acetylation [30–32] that may affect their location or activity.

This complex regulation allows the correct progression of the splicing process, including the variations that cause alternative splicing. Specifically, there are five different types of alternative splicing: 1) cassette exon skipping, an exon is excluded together with the two flanking introns; 2) mutually exclusive exons, two exons that cannot be included together, one of each is excluded in two different isoforms; 3) intron retention, there is no cutting in the intron resulting in its inclusion in the mature RNA; 4) alternative 3’ splice site and 5) alternative 5’ splice site, the exon is cut in a different site thus it is not fully included in the final RNA [24, 33, 34] (Fig. 1B). Taken together, all this information demonstrates the great complexity of the splicing process and underscores its relevance in controlling the normal functions of the cell.

## Splicing is altered in cancer

The precise understanding of the molecular mechanisms underlying the process of alternative splicing has lagged behind that of other fundamental processes, such as transcription or translation. However, evidence was soon found to suggest that alterations in the splicing process were associated with human diseases, and particularly to tumor development and cancer [35]. Actually, there is now ample consensus that dysregulated splicing, originated by mutations or changes in the splicing machinery and/or the abnormal profile of splicing events, is a key phenomenon contributing to all cancer types studied so far, and thus dysregulated splicing participated in all cancer hallmarks, as has been recently reviewed in detail [36, 37]. Nevertheless, while the altering of splicing can be regarded as a common feature in cancer, the specific changes that it involves are specific for each tumor type and, therefore, should be precisely examined in detail in the appropriate samples and representative experimental models. Accordingly, in the present review, we have focused on the dysregulation of splicing in pancreatic cancer.

## Splicing dysregulation in PDAC

**Fig. 2:**
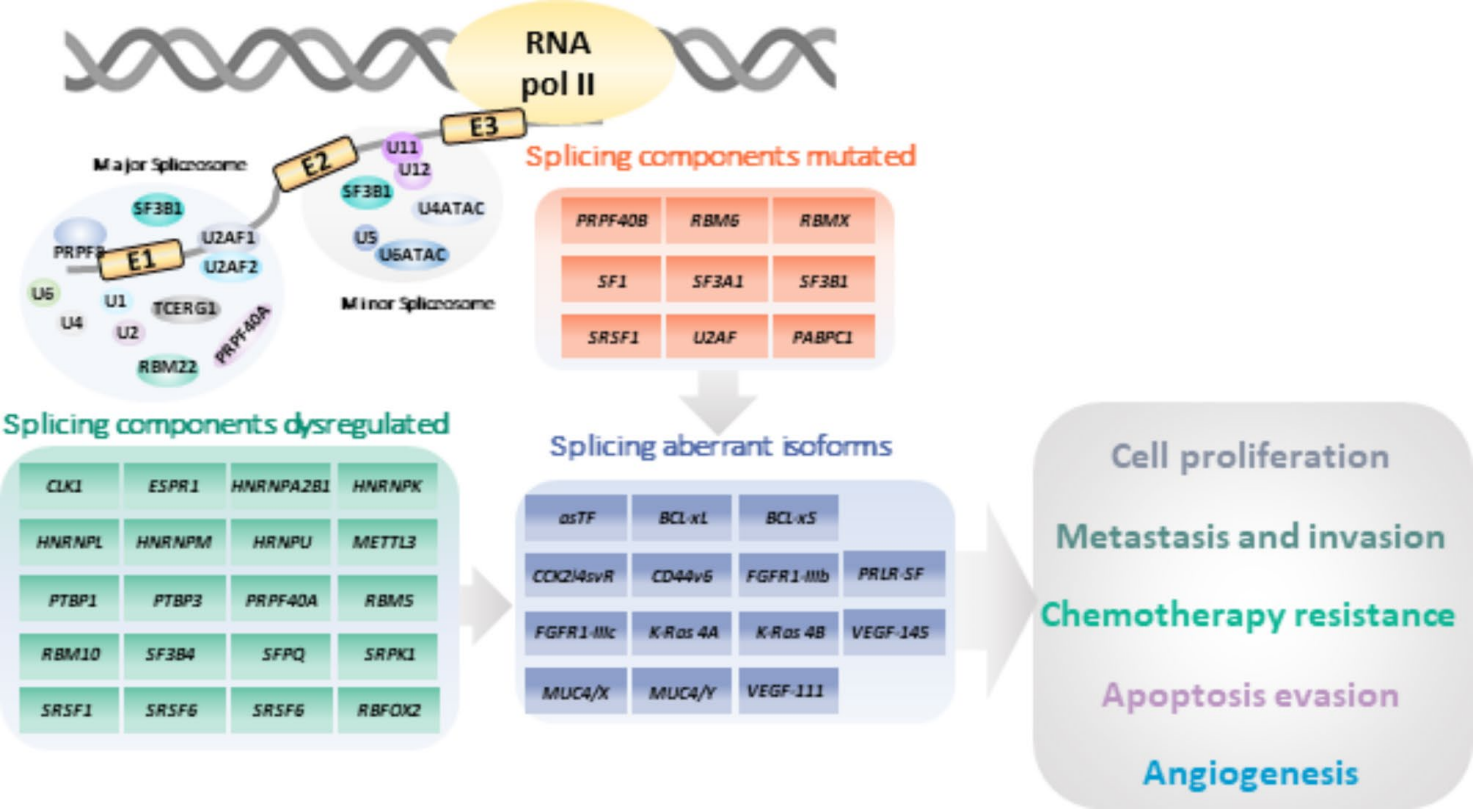
Splicing alterations in PDAC. Splicing components have been shown to be mutated and their expression dysregulated in PDAC, leading to the disequilibrium in the isoforms or the appearance of aberrant isoforms that cause or promote several cancer features

Despite the impressive growing list of alterations in genes and regulatory mechanisms that have been described to date, these are still insufficient to provide an effective therapeutic strategy to battle PDAC [12]. In this regard, an increasing number of studies indicate that the dysregulation of splicing can play an important role in PDAC progression. Such dysregulation may arise from mutations or alterations in the expression levels of specific components of the splicing machinery. Additionally, alterations in the relative proportions of splice variants, and even the emergence of aberrant variants, might contribute to the intricate landscape of PDAC development (Fig. 2). In the following subsections we will discuss about the alterations in the splicing process that have been described in PDAC.

### Dysregulation of splicing machinery components

Alterations in the expression of components of the molecular machinery that operate and control the splicing process have already been described in an extensive list of diseases, including many tumor pathologies [35, 38–40]. In PDAC, pioneering studies by Carrigan et al. evaluated expression levels of selected genes in human pancreatic cancer cell lines, discovering a downregulation of 30% of spliceosomal genes, revealing a clear repression of splicing machinery components [41]. More recently, Wang et al. validated some of these changes in PDAC human samples, establishing an expression signature of the spliceosome and splicing regulatory genes that discriminated with high accuracy between tumor and healthy samples [42]. Interactions and implications of alternative splicing in PDAC pathogenesis have been subsequently reviewed [43], emphasizing the promising prospect of dissecting its role in this pathology.

Further work has provided a deeper understanding of the dysregulations in the expression of the splicing machinery in PDAC, revealing that they frequently consist in an altered expression of spliceosome components and/or splicing factors (Table 1), which usually leads to an imbalanced profile of splice variants and/or the appearance of aberrant variants. Considering that the correct functioning of the splicing process regulates the overall balance of RNA variants in the cell, it is not to be unexpected that changes in the expression of splicing-related proteins could dramatically modify cell homeostasis, including key processes in PDAC evolution. This is the case of the overexpression of splicing machinery components that are associated with proliferation and apoptosis, such as *SRPK1* [44, 45], *CLK1* [46], *HNRNPK* [47], *PTBP3* [48], *HNRNPL* [49], *HNRNPA2B1* [50] and *ESRP1* [51]; with metastasis and invasion, such as *SF3B1* [52], *CLK1* [46], *PRPF40A* [53], *ESRP1* [51], *SRSF6* [54] and *RBFOX2* [55]; with the acquisition of chemotherapy resistance, such as *SRPK1* [44, 45], *SRSF1* [56], *PTBP1* [57] and *SRSF3* [58]; with autophagy, such as *SFPQ* [59] and *PTBP3* [48]; with ubiquitination and degradation of some proteins, such as *HRNPU* [36]; or with the activation of relevant pathways in PDAC, such as the signaling mediated by *KRAS*, driven by *HNRNPK* [47], or the MAP kinases pathway by *METTL3* [60], or the pancreatitis and KRASG12D-mediated cancer promoted by *SRSF1* [61].

**Table 1:** Dysregulations in the expression of the splicing machinery in PDAC

Splicing Machinery	Dysregulation	Variants	Functional role	Ref No.
***CLK1***	Upregulated	*METTL14* ^*exon10*^ *Cyclin L2* ^*exon6.3*^	*Growth and metastasis, and regulation of m6A methylation*	[46]
*(CDC Like Kinase 1)*				
***ESRP1***	Upregulated	*FGFR-2 IIIb* *FGFR-2 IIIc*	*Cell growth, migration, invasion, and metastasis*	[51]
*(Epithelial Splicing Regulatory Protein 1)*				
***HNRNPA2B1***	Upregulated	*Bcl-x(s)* *Bcl-x(L)*	*Apoptosis, proliferation, and metastasis*	[50]
*(Heterogeneous Nuclear Ribonucleoprotein A2/B1)*				
***HNRNPK***	Upregulated	*GTPase Activating Proteins*	*Tumor growth and sensibility to spliceosome inhibitors*	[47]
*(Heterogeneous Nuclear Ribonucleoprotein K)*				
***HNRNPL***	Upregulated		*Migration and epithelial mesenchymal transition*	[49]
*(Heterogeneous Nuclear Ribonucleoprotein L)*				
***HNRNPM***	Downregulated		*Adaptation to a hypo-vascular environment*	[64]
*(Heterogeneous Nuclear Ribonucleoprotein M)*				
***HNRNPU***	Upregulated		*Putative biomarker*	[36]
*(Heterogeneous Nuclear Ribonucleoprotein U)*				
***METTL3***	Upregulated		*Mitogen-activated protein kinase cascades, ubiquitin-dependent process, and RNA splicing*	[60]
*(Methyltransferase Like 3)*				
***PTBP1***	Upregulated	*PKM*	*Drug resistance*	[57]
*(Polypyrimidine Tract Binding Protein 1)*				
***PTBP3***	Upregulated		*Drug resistance*	[48]
*(Polypyrimidine Tract Binding Protein 3)*				
***PRPF40A***	Upregulated		*Putative biomarker*	[53]
*(Pre-MRNA Processing Factor 40 Homolog A)*				
***RBM5***	Downregulated		*KRAS expression, lymph node and distant metastases, stage, and nerve and venous invasion*	[65]
*(RNA Binding Motif Protein 5)*				
***RBM10***	Upregulated	*TERT*	*Telomere shortening*	[62]
*(RNA Binding Motif Protein 10)*				
***SF3B1***	Upregulated	*BCL-XS/BCL-XL* *KRASa/KRAS* *Δ133TP53/TP53*	*Tumor grade, lymph node involvement*	[52]
*(Splicing Factor 3b Subunit 1)*				
***SF3B4***	Downregulated		*Cell growth, proliferation, and migration*	[63]
*(Splicing Factor 3b Subunit 4)*				
***SFPQ***	Upregulated		*Autophagy and cell growth*	[59]
*(Splicing Factor Proline And Glutamine Rich)*				
***SRPK1***	Upregulated		*MAPK and AKT signaling modulation*	[45]
*(SRSF Protein Kinase 1)*				
***SRSF1***	Upregulated	*MNK2b*	*Drug resistance*	[56]
*(Serine And Arginine Rich Splicing Factor 1)*				
***SRSF3***	Upregulated	*CDKN2B-AS1*	*Drug resistance*	[58]
*(Serine And Arginine Rich Splicing Factor 6)*				
***SRSF6***	Upregulated		*Epithelial-to-mesenchymal transition, metastasis*	[54]
*(Serine And Arginine Rich Splicing Factor 6)*				
***RBFOX2***	Downregulated	*RHO GTPase pathways*	*Metastasis, cytoskeletal organization and focal adhesion formation*	[55]
*(RNA Binding Fox-1 Homolog 2)*				

Although current evidence supports that overexpression of splicing machinery components is more frequent in PDAC than initially observed, there are numerous examples where such components are repressed, indicating a putative tumor suppressor role. For example, *ESRP1* regulates the expression pattern of *FGFR-2* isoforms, attenuating cell growth, migration, invasion, and metastasis in PDAC cell lines [51]. Likewise, *SRSF6* hinders pancreatic cancer cells migration and invasion by regulating *ECM1* alternative splicing isoforms [54]. Further, *RBM10* promotes the appearance of the *TERT* splicing isoform *TERT-S*, which in contrast with *TERT-FL*, is not able to maintain telomeres [62]. *SF3B4* inhibits the growth and migration of cancer cells preventing STAT3 phosphorylation [63]. In the same line, downregulation of *HNRNPM* and *RBM5* is associated with an increase in tumor aggressiveness. Specifically, *HNRNPM* is implicated in the adaptation to a hypovascular environment [64]; while *RBM5* expression inversely correlates with *KRAS* levels and is associated with clinicopathological features and appears to promote tumor progression [65]. Splicing factor *CELF2* is downregulated in PDAC and associated to PDAC progression, where its downregulation affects the splicing pattern of *CD44*, thereby regulating endoplasmic reticulum-associated degradation [66].

### Mutations in splicing factors in PDAC

Mutations in genes specifically involved in the splicing process are increasingly recognized as a source of pathological effects in a range of diseases, including cancer [17, 35]. These types of mutations are particularly frequent in pathologies such as acute myeloid leukemia or myelodysplastic syndromes, where they are tightly linked to etiology and offer therapeutic opportunities [67–69]. Whole-exome sequencing of PDAC has revealed a number of mutations in key oncogenes and tumor suppressor genes, as *KRAS* or *TP53* [70], which are known to be among the most commonly mutated genes in PDAC [4]. Nonetheless, those studies also identified mutations, although with lower frequency, in genes involved in other essential processes including splicing (Table 2), which conferred a higher tumor heterogeneity in PDAC. Genomic studies have also identified recurrent mutations affecting the early components of the RNA splicing machinery, such as *SF3B1* [6]. Mutations in *SF3B1* are the most common across multiple tumor types, mainly found in myelodysplastic syndrome [71, 72] and other hematologic malignancies, uveal melanoma and breast cancer [73, 74], where its high mutational frequency altered the capacity of the spliceosome to recognize the pre-RNA pattern [25, 75]. *SF3B1* mutations are generally heterozygous, major hotspots identified matched with codon positions K700 and R625, which correspond with deleterious mutations [76–78]. However, *SF3B1* mutations differ between each pathology, suggesting context-depending functional differences. For example, it has recently been described that *SF3B1* K700E mutation increased glycolysis and the Warburg effect in PDAC, by promoting aberrant splicing of *PPP2R5A* and therefore activating c-Myc signaling, a positive regulator of glycolysis [79]. Thus, while the knowledge on the effects of *SF3B1* mutations in PDAC is still limited, it is worth pointing out that several such mutations have been identified in a small but appreciable percentage of patients, such as G740E, N763S, K843R [80], P342T [7], K700E, L773R [6], K700E, Q699_K700delinsHE, N763S, K741K [81].

**Table 2:** Splicing machinery mutations found in PDAC

Splicing Machinery	Mutation	References
***SF3B1*** *(Splicing Factor 3b Subunit 1)*	P342T L415P R625C H662Q K666R Q699_K700delinsHE K700E N763S K843R L773R K741K K946T	7 160 158 158 159 81 6, 74 81 80 6 81 160
***SF3A1*** *(Splicing Factor 3a Subunit 1)*	S58I	7
***U2AF1*** *(U2 Small Nuclear RNA Auxiliary Factor 1)*	A47V S34F	81 81
***U2AF2*** *(U2 Small Nuclear RNA Auxiliary Factor 2)*		81
***SRSF1*** *(Serine And Arginine Rich Splicing Factor 1)*		82
***PRPF40B*** *(Pre-MRNA Processing Factor 40 Homolog B)*	L265M	7
***SF1*** *(Splicing Factor 1)*	R380Q R662* Q269_P273del	81 81 81
***RBM6*** *(RNA Binding Motif Protein 6)*		161
***PABPC1*** *(PolyA Binding Protein Cytoplasmic 1)*	M158Nfs*8 D165G E345* E345K L562S T319I P402L F335Lfs*19I R475Q R481C	74 4 4 4 157 7 7 7 158 158
***RBMX*** *(RNA Binding Motif Protein X-Linked)*	D312N P106Ffs*32 A78T R341W	6 6 4 157

In line with the findings on *SF3B1*, mutations have also been described in other splicing factors like *U2AF2*, which is involved in pre-RNA *branch site* (BS) binding; *SRSF1*, a SR protein that generally promotes exon inclusion [42, 82]; *PABPC1*, required for poly(A) shortening, the first step in RNA decay [4, 83]; or *RBMX*, a RNA-binding protein that plays a crucial role in alternative splicing of several pre-RNAs [84].

Collectively, these studies provide compelling evidence that mutations and particularly altered expression in specific components of the splicing machinery are a common feature in PDAC, and that such dysregulations often result in pathological consequences. These observations also point to the aforementioned alterations as potential targets for therapeutic intervention, which is already being exploited via diverse strategies, as discussed below. Nevertheless, the splicing machinery, which integrates all spliceosome components and their associated splicing factors, is extraordinarily complex and there is still much to be learned on its precise expression and regulation in PDAC. A comprehensive understanding of its abnormal functioning will likely help to better comprehend the development and progression of PDAC and will facilitate the discovery of new molecular targets and tools.

### Alterations in splicing variants in PDAC

Defects in alternative splicing often result in the appearance of abnormal splicing variants that can play an oncogenic function by conferring advantages to cancer cells. Observations from early studies on alternative splicing in PDAC, employing expression microarray techniques, prompted further analysis that applied more sophisticated bioinformatic approaches to explore the pattern of splicing events and signatures in PDAC cell lines [41] and human tissue [42, 85]. The landscape of alternative splicing in PDAC shows that the most common alterations in the protein-coding genes are skipped exon and alternative first exon, followed by intron retention [42].

In the last two decades, numerous studies have aimed to achieve a deeper understanding of the precise regulation of alternative splicing of individual genes and its mechanistic basis and pathological implications in PDAC (Table 3). A case as paradigmatic as intricate is the study of *CD44*, a multifunctional cell surface glycoprotein involved in structural and functional roles in cell-cell and cell-matrix interactions. The standard isoform of *CD44* (*CD44s* or *CD44h*) only contains the five first exons [1–5] and last five exons [16–21], while the alternative variants CD44v have variable exons (v1-v10) that are alternatively spliced and incorporated between the exons 5–16, conditioning its final structure and thus its biological role [86]. The *CD44* variants *CD44v2* and *CD44v6*, can be detected in human PDAC tissue by immunohistochemistry, where their expression is connected to an increase in mortality rate [87–89]. Recently, Zhao et al. delved into the potential role of *CD44* isoforms in PDAC cell lines, linking these to an EMT phenotype and higher invasiveness and chemoresistance features [90]. Another study by Zhu et al. described that *CD44v3* is associated with poor prognosis in PDAC, with its generation being regulated by splicing factor *U2AF1* [91]. A study more focused on metastasis by Xie et al. showed that isoform *CD44v6* is essential for liver fibrosis and metastasis from PDAC and could be used as metastasis and prognosis biomarker [92]. Actually, it was demonstrated that peptide inhibitors of this isoform block tumor growth and metastasis in rodent models of PDAC [93]. Thus, although it is still unknown whether and how expression of *CD44* variants specifically affect the cellular function of PDAC cells in vivo in patients, alternative splicing of this gene and their variant products comprise likely actionable targets and tools in PDAC.

**Table 3:** Altered or aberrant isoforms in PDAC

Gen	Splicing Isoform	Mechanism	References
*CD44*	*CD44v2* *CD44v6*	EMT to MET transition and tumor invasiveness	90
*CCK2*	*CCK2i4svR*	Cholecystokinin and gastrin mediated pathways	97
*PRLR*	*PRLR-SF*	Proliferation, tumor growth	101
*FGFR1*	*FGFR1-IIIb* *FGFR1-IIIc*	Cell proliferation, adhesion, and movement	107
*MUC4*	*MUC4/Y* *MUC4/YX*	Cell adhesion, immune response, and cell signaling	110
*BCL2L1*	*BCL-xL* *BCL-xS*	Apoptosis	114
*TF*	*flTF* *asTF*	Blood coagulation cascade and vascularization	118
*VEGFA*	*VEGF-111* *VEGF-145*	Transition from other lesions to PDAC	122
*KRAS*	*KRAS4A* *KRAS4B*	Cell proliferation and apoptosis	124

Splicing variants of relevant receptors have also been described in PDAC. An aberrant variant of secretin receptor (encoded by the *SCTR* gene) was found, where the third exon is spliced out and therefore residues 44–79 from the NH [2]-terminal tail are eliminated, blocking secretin binding, and thus prompting tumor growth and progression [94, 95]. Moreover, the potential of the secretin receptor variant as an early diagnostic serum biomarker has been proposed [96]. Likewise, an aberrant variant of the cholecystokinin and gastrin receptor, *CCKR2*, which retains the fourth intron, was identified in PDAC, but was absent in normal pancreas. This variant is constitutively active, may contribute to pathological features in vitro, and it might be associated to a polymorphism of *U2AF35*, affecting the splicing regulation of this receptor [95]. Furthermore, Ryberg and collaborators reported three novel *CCKR2* splice-forms in PDAC, different from the better known *CCK2i4svR* variant, which might have similar functions [97]. Another example is the prolactin receptor, *PRLR*, that has been previously linked to carcinogenesis [98]. This gene undergoes alternative splicing in PDAC, allowing for the formation of several splicing isoforms that differ from each other in the intracellular domain and thus they promote the activation of different downstream signaling pathways [99]. The most abundant and best-known isoform is *PRLR-LF*, by which prolactin mainly transmit its signals. In contrast, the short isoform, *PRLR-SF*, is not as well understood. A recent study demonstrated that *PRLR-SF* reduces nucleotide synthesis by inhibiting the pentose phosphate pathway (PPP) through the NEK9-Hippo pathway in PDAC cells and in xenografted tumors in mice, hindering proliferation and tumor growth. *PRLR-SF* regulates the PPP pathway by reducing the expression of two rate-limiting enzymes G6PD (Glucose-6-phosphate dehydrogenase) and TKT (transketolase). PPP generates both pentose phosphate for nucleic acid synthesis and NADPH for fatty acid synthesis, being a key pathway for cell proliferation which is also upregulated in PDAC cancer stem cells (CSCs) [100]. Therefore, *PRLR-SF* might play an important role in metabolic reprograming, thus preventing PDAC tumor progression [101].

Fibroblast Growth Factor Receptors comprise a family of tyrosine kinase receptors (*FGFR1-4*), whose presence, signaling, and therapeutic potential in PDAC has been recently reviewed [102]. Early work demonstrated the existence of different splicing isoforms for *FGFR1*, *2* and *3* in PDAC [103], where their differential expression was linked to tumor biology. Thus, whereas *FGFR1-IIIb* and *FGFR1-IIIc* isoforms are mainly expressed in epithelial and mesenchymal cells, respectively, they are co-expressed in PDAC cells, promoting tumorigenicity by modulating cell proliferation, adhesion, and movement, possibly via activation by FGF5 [104–108].

Also related with epithelial cells are mucins, which protect and lubricate the ducts and are involved in the differentiation and renewal of the epithelium and the modulation of cell adhesion, immune response, and cell signaling. The expression of mucin subtypes and their splice variants are used to classify PDAC in four different subtypes, which were differentially associated to patient survival [109]. Specifically, *MUC4* presents several splicing variants, differing in the lack of exons 0–21, being *MUC4/Y* and *MUC4/X* [110] the best known isoforms. Interestingly, *MUC4* isoforms are mainly expressed in PDAC and correlated with tumor malignancy, while the canonical isoforms are not detectable in normal pancreas [111–113].

The BCL2 family is composed by a number of proteins that play critical roles in apoptosis. Alternative splicing of one of its members, *BCL2L1*, results in the production of two variants with opposite functions, *BCL-xL* (anti-apoptotic) and *BCL-xS* (pro-apoptotic), due to the retention/lack of exon 2, respectively. In PDAC, *BCL2* dysregulation has been associated with apoptosis resistance, due to *BCL2L1* anti-apoptotic isoform overexpression in human tumor tissue [114]. Furthermore, its expression has been related to the progression to high-grade PanINs in mice [115]. The presence of specific *BCL2* isoforms is differentially regulated by several splicing factors, where *HNRNPF*, *HNRNPH*, *KHDRBS1*, *RBM11*, or *RBM25* promote the short variant, whereas *SRSF1*, *SRSF9*, or *SF3B1* promote the longer variant [116]. In PDAC, this regulation has been examined in model cell lines, where the role of *SF3B1* in the 5’splice site activation of *BCL-xS* has been confirmed [52, 117].

Tissue Factor (*TF*) is a glycoprotein primarily involved in the blood coagulation cascade. Its alternative splicing isoform, known as *asTF*, excludes the fifth exon and exhibits low prothrombogenic potential [118]. Regulation of *asTF* has been linked with several splicing factors, specifically with the SR family: *SRSF6*. In PDAC, *asTF* has been identified in tumor tissue, correlating with tumor infiltration. Moreover, in PDAC cell lines, *asTF* promotes tumor vascularization and tumorigenesis by different pathways, such as EGFR and EMT [119]. Likewise, asTF plasmatic levels are found at higher levels in PDAC patients than in healthy subjects, suggesting a potential use as a biomarker [120].

The vascular endothelial growth factor A (*VEGFA*) gene has 8 exons that can be alternatively spliced in multiple ways. Isoforms generated differ from each other in their affinity for binding sites, tissue localization, and their capacity to be diffusible [121]. *VEGFA* was previously described as a potential biomarker for benign pancreatic serous cystic neoplasm, which could help differentiate them from other types of lesions that can evolve into PDAC, like intraductal papillary mucinous neoplasms and mucinous cystic neoplasms [121]. Recently, it was observed that *VEGFA* spliced isoforms show different expression levels in normal pancreas, benign pancreatic serous cystic neoplasms, mucinous cystic neoplasms and intraductal papillary mucinous neoplasms [122].

*KRAS* mutations are prevalent in more than 95% of pancreatic cancers [123]. The *KRAS* gene encodes two splicing isoforms, *KRAS4A* and *KRAS4B*, products of alternative splicing of the fourth coding exons 4 A and 4B, being mutually exclusive. While *KRAS4B* is one of the most studied oncogenes, the role of *KRAS4A* is much less known. In PDAC, both isoforms are detectable, but their specific role has not yet been elucidated [52], although it may parallel that found in colorectal carcinoma, where *KRAS4A* has been associated to a suppressive and pro-apoptotic activity, while *KRAS4B* would play an anti-apoptotic effect [124].

Study of EMT-related alternative splice events unveiled that specific splice events from *TMC7* and *CHECK1* were associated with metastatic PDAC. Additionally, the inclusion of exon 17 of *TMC7* was associated to poor prognosis in PDAC, meanwhile its knockdown reduced tumor-related properties [125]. In addition, aberrantly spliced variants have also been described and their role examined in PDAC. For example, while searching genomic variants that could be linked to splicing alterations, an allele was found to promote the generation of a truncated splice variant of the Elongator Acetyltransferase Complex Subunit 2 (*ELP2*). This aberrant variant acts as tumor suppressor for PDAC by blocking STAT3 oncogenic pathway [126].

As the precision and breadth of RNA sequencing approaches improve and the analysis of splicing receives more attention, the discovery of novel variants and the study of their potential pathogenic role advances further in PDAC will certainly heighten.

## Splicing modulation for therapeutic benefit

**Fig. 3:**
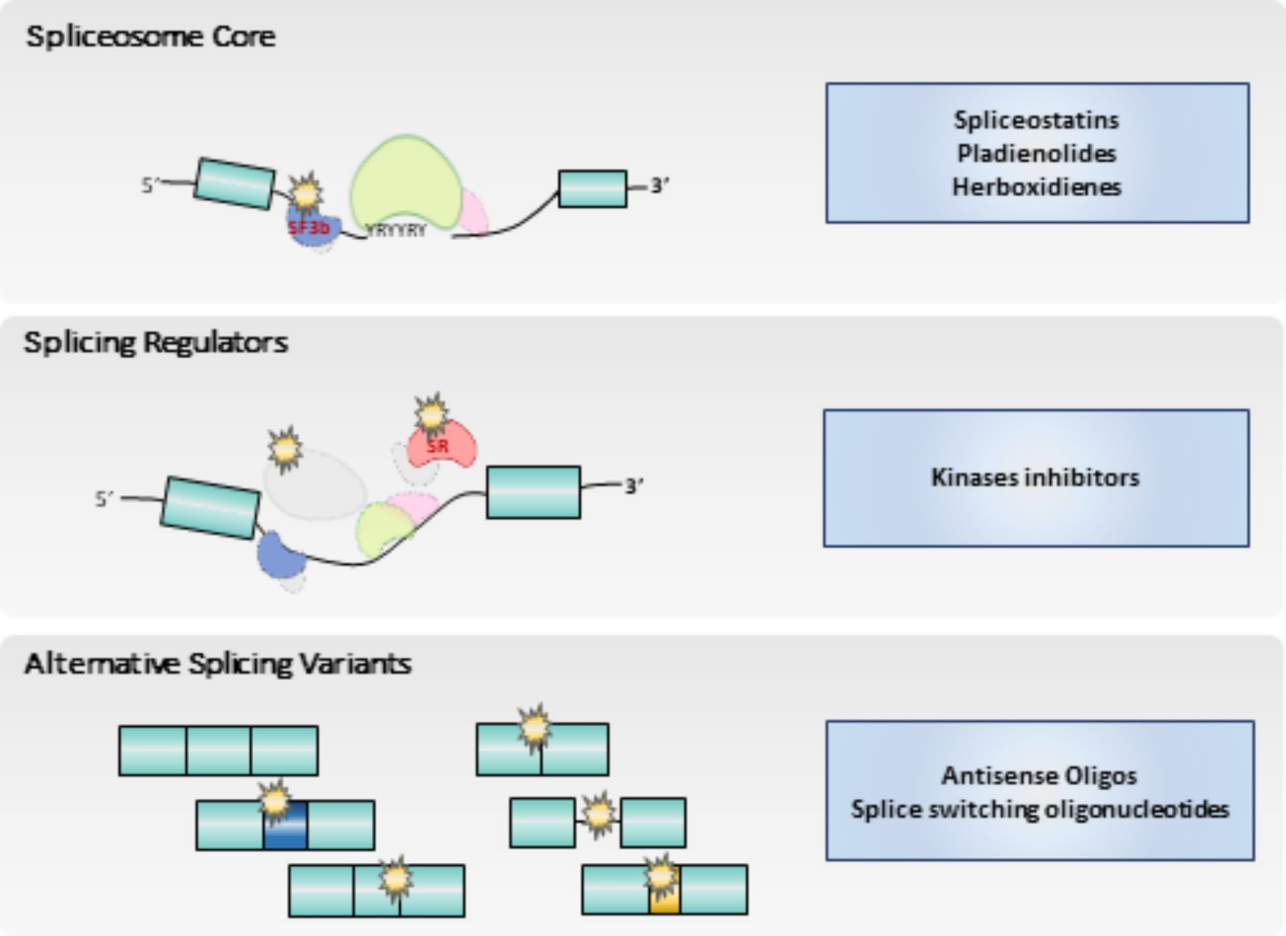
Splicing components as therapeutic targets. A number of techniques have been developed to chemically regulate splicing, in order to avoid the harmful effect of its dysregulation. Those techniques include chemical inhibitors of the core (top panel), regulators of splicing factors, like kinases inhibitors (middle panel), and oligonucleotides that bind to regulator sequences (bottom panel)

It is now widely accepted that splicing alterations can play important roles in the development and progression of cancer. However, splicing dysregulation can also influence tumor treatment response, as certain aberrant variants modify cellular functions leading to chemotherapy and targeted therapy resistance. Nonetheless, the pathogenic role of altered splicing also provides novel opportunities to tackle cancer, by designing and developing strategies focused on counteracting the effects of splicing errors and employing splicing dysregulation as an actionable therapeutic target. The following are some of the strategies that are being currently applied (Fig. 3).

### Targeting the splicing core

The widespread alteration of splicing in cancer, among other pathologies, has prompted the development and testing of different types of molecules capable of interacting with specific elements of the spliceosome core and modulating their functioning. Three of these compounds, representative of different chemical natures, are Spliceostatins, Pladienolides and Herboxidienes. Some of these share similar mechanisms aimed at inhibiting SF3B1 and, consequently, interfering with the RNU2 complex, destabilizing it and preventing the transition of the spliceosome complex. SF3B1 is known to physically interact with HIF1α and induce hypoxia, promoting PDAC malignancy [127]. The potential clinical utility of these SF3B1 targeting molecules and their derivates has been demonstrated in several studies. E7107 compound, a Pladienolide B derivative [128], and H3B-8800 [129], a SF3B complex modulator able to kill spliceosome-mutant epithelial and hematologic tumor cells, have been tested in preclinical assays and are currently under clinical evaluation in a phase I study (NCT02841540). In fact, Pladienolide B has been tested in a preclinical PDAC study, demonstrating its capability to decrease multiple cancer features in cells, zebrafish, and mice models by altering relevant signaling pathways and splicing events, and reducing CSC stemness, making CSCs more sensitive to chemotherapy treatment [52]. However, further clinical trial efforts will be required to confirm the toxicology, safety, and potential benefit of compounds targeting the splicing machinery.

### Targeting splicing regulatory elements

An alternative approach to manipulate and/or reverse splicing alterations, without blocking the spliceosome machinery core, is based on targeting regulatory proteins that modulate splicing. The use of these splicing regulators could be directed to mutated or altered molecules involved in pathological processes.

Phosphorylation and dephosphorylation of proteins by kinases represent a pivotal regulatory mechanism for multiple biological processes like metabolism, transcription, cell cycle progression, cell movement, apoptosis, and differentiation [130–133]. Thus, the potential of kinases as therapeutic targets has received considerable attention. Alternative splicing is also regulated by kinases that phosphorylate/dephosphorylate splicing factors, like the SR proteins [134], serving as a signal of nuclear localization and facilitating interaction with other splicing factors. This phosphorylation can be performed by SR protein kinases (SRPKs), topoisomerase 1 (TOP1), protein kinase B (PKB/AKT), NIMA-related kinases (NEK2), PRP4 kinase (PRP4K), dual-specificity tyrosine phosphorylation-regulated kinase 1 A (DYRK1A) [135, 136], cAMP-dependent protein kinase (PKA) [137, 138], or by the family of cdc-like kinases (CLKs) [139]. Thus, over the last decade, an increasing number of studies have revealed that dysregulation of splicing kinases has an important role in tumorigenesis and therapeutic response [140], as it is the case of SM08502, which has been shown to reduce Wnt pathway signalling by inhibiting CLK activity and SR family phosphorylation, leading to the disruption of the spliceosome activity. A Phase 1 clinical trial has been launched to evaluate the safety and pharmacokinetics of orally administered SM08502 in patients with advanced solid tumors [141].

Altogether, these studies emphasize the great potential of modulating splicing regulators for the treatment of cancer. Interestingly, these drugs are already being tested in clinical trials, particularly on oncohematological diseases, also in certain solid tumors. Of particular interest, Bromodomain and Extra-Terminal motif, BET, protein family inhibitors, such as ZEN-3694 are currently evaluated in triple negative breast cancer (clinical trial no. NCT03901469), and metastatic castration-resistant prostate cancer (NCT02705469, NCT02711956) [142]; and OTX015, which was proposed to be used in solid tumors (NCT02259114), and the results on leukemia and lymphoma have been reported [143, 144]. Additionally, there are also core spliceosome inhibitors, such as the case of H3B-8800, a Pladienolide-B derived SF3B1 inhibitor, which specifically targets cancer cells harboring splicing factor mutations [129], which is currently being tested on leukemia and myelodysplastic syndromes, MDS, (NCT02841540) [145]. GSK3368715 is a PRMT1 inhibitor, which regulates numerous nuclear ribonucleoproteins involved in the splicing process [146], and has been used in solid tumors and diffuse large B-cell lymphoma (NCT03666988). Similarly, the PRMT5 inhibitors, JNJ-64619178, GSK3326595 and PF06939999, modify snRNP-associated Sm proteins [147] and are currently being used in solid tumors, non-Hodgkin lymphoma and MDS (NCT03573310, NCT03614728/NCT02783300/NCT04676516 and NCT03854227, respectively). In the same line, the anti-cancer sulfonamide compound E7820, which degrades the splicing factor RBM39 is being assessed in MDS, acute myeloid leukemia (AML), or chronic myelomonocytic leukemia (CMML) (NCT05024994), which have shown acceptable safety [148]. Moreover, the ATR inhibitor Ceralasertib is being tested in MDS and CMML (NCT03770429), although it does not directly affect splicing, those patients harboring splicing factor mutations are more sensitive to this treatment [149]. Despite the promising results of these clinical trials, the use of splicing inhibitors in PDAC has not yet been reported.

### Oligonucleotides

Another promising approach to target defects and alterations related to the splicing process is based on the use of short antisense oligonucleotides (ASO), which act by blocking the interaction between proteins and RNAs or between two RNAs. Splice-switching antisense oligonucleotides (SSOs) are nucleotides composed by 15–30 synthetic nucleotides or analogues, chemically modified to avoid enzymatic degradation of the target RNA, which are able to bind specifically to a target complementary sequence and thereby block the binding between splicing factors and pre-RNA [150]. Oligonucleotide therapy has already been approved by the FDA to treat certain diseases, such as Spinal Muscular Atrophy [151]. Their use in cancer is under evaluation [152], after promising preclinical studies showing their potential in various tumor pathologies [153, 154].

Regarding PDAC, pre-clinical in vitro studies showed that OGX-427, an antisense nucleotide complementary to *HSP27* (Heat shock protein 27), inhibits proliferation, induces apoptosis, and enhances gemcitabine chemosensitivity in the MIAPaCa-2 PDAC cell line [155]. In fact, a phase II clinical trial in patients with metastatic pancreatic cancer was conducted to prove the efficacy of OGX-427 (Apatorsen; NCT01844817), comparing its effect plus/either gemcitabine/nab-paclitaxel compared to placebo. Despite pre-clinical data showing efficacy in pancreatic cancer cell lines, the addition of Apatorsen to chemotherapy did not improve outcomes in this clinical trial [156]. This and other related studies support the feasibility of using ASO as a tool to modulate splicing-related defects in PDAC, and in fact, there is experimental work underway in this direction.

## Conclusion and outlook

PDAC is a highly lethal cancer, often diagnosed at advanced stages. Despite considerable research efforts to find novel therapies, groundbreaking advances remain elusive. The aim of this review was to emphasize the molecular complexity of alternative splicing and the relevance of its alterations in PDAC. Recent studies have revealed significant mutations and alterations in the expression levels of key components of the splicing machinery and splicing variants in PDAC. These alterations are tightly associated with pivotal clinical and molecular features of tumor development or progression.

Future studies should aim to achieve a more comprehensive and precise understanding of splicing and its related constellation of molecular components and machineries. To achieve this goal, newly developed technical and methodological strategies should be applied enabling to dissect and modulate splicing components in appropriate models. This approach will leverage this field as an alternative source of omics data to identify new biomarkers and to uncover previously hidden therapeutic targets. Also, the recent use of novel approaches to target splicing (e.g., anti-splicing drugs and oligonucleotides) will likely increase the number and potential extent of therapeutic opportunities.

However, to date, most available studies on this subject have been conducted using established cell lines or resectable tumors. Consequently, they may not fully represent the actual altered spliceosomic landscape in the most vulnerable population of metastatic patients, in whom splicing likely undergoes a more advanced mutational evolution from primary to metastatic tumors. Thus, further studies should focus on this metastatic patient population, through the analysis of metastatic tumor biopsies or with circulating tumor cells (CTC, a useful surrogate model of the metastatic tumor) allowing to examine differences with the primary tumors. These studies may require the use of Patient-Derived Xenografts (PDXs), CTC-derived PDXs, and organoids from metastatic biopsies to address methodological challenges.

Ultimately, the overarching goal is to understand the mechanistic underpinnings of the splicing machinery and dissect its resulting outcomes, the splicing variants. This will allow to obtain a fully detailed spliceosomic landscape in PDAC, and this information may potentiate the development of innovative tools and provide novel avenues to tackle this incurable cancer.

## Data Availability

Not applicable.

## Abbreviations

PDAC: Pancreatic ductal adenocarcinoma
IPMN: Intraductal papillary mucinous neoplasm
MCN: Mucinous cystic neoplasm
ITPN: Intraductal tubulopapillary neoplasm
PanIN: Pancreatic intraepithelial neoplasia
FOLFIRINOX: Folinic acid, 5-fluorouracil, irinotecan, and oxaliplatin
snRNAs: Small nuclear RNAs
snRNP: Small nuclear ribonucleoproteins
mRNA: Messenger RNA
ESE: Exonic splicing enhancer
ISE: Intronic splicing enhancer
ESS: Exonic splicing silencer
ISS: Intronic splicing silencer
miRNA: Micro-RNA
SR protein: Serine-arginine protein
CSC: Cancer stem cell
EMT: Epithelial mesenchymal transition
ASO: Antisense oligonucleotide
SSO: Splice-switching antisense oligonucleotide
FDA: Food and drug administration
CTC: Circulating tumor cell
